# Effect of listening to music among patients with dental anxiety

**DOI:** 10.6026/973206300200074

**Published:** 2024-01-31

**Authors:** K Ponni, Sivakumar Dhandapani, A Pravin Kumar, Vashni Solomon, B Anselm Justhius Fabi, M Vennila

**Affiliations:** 1 Government Chengalpattu Medical College and Hospital, Tamilnadu, India; 2 Government Medical College & ESIC Hospital Coimbatore, Tamilnadu, India; 3 Government Arignar Anna Memorial Cancer Hospital & Research Institute, Tamilnadu, India; 4 Government Villupuram Medical College and Hospital, Tamilnadu, India

**Keywords:** Dental anxiety, music listening

## Abstract

Listening to music during dental treatment is widely accepted as a relaxation method. However, its effects are unclear on the psychological and vital parameters. Patients who came to the Government Hospital of 18 - 60 years old, medically fit and indicated for dental extraction and exhibiting dental anxiety were included in the study. Music was not played in the control group (n=100) whereas in the experimental group (n=100), music was played according to patients' preference under the genre western, classical, or popular. Blood pressure, Body temperature, Pulse rate, Oxygen saturation and Respiratory rate were recorded. Results showed no statistical difference between the control and experimental group except the respiratory rate which increased statistically in both control and experimental group. Thus, the current study reveals that the effect of music over an invasive procedure like extraction that has been perceived as painful treatment for a long time has little effect on adult patients.

## Background:

Dental anxiety is a frequently encountered problem, which has been cited as the fifth-most common cause of anxiety by Agras *et al*. [1]. Prevalence of dental anxiety among Indians is high when compared to that of western countries [2,3,4]. Anticipation of perceived or actual physical risk in an unfamiliar environment like hospital, loss of control, dependence on strangers, and separation from friends and family are some of the factors that can contribute to the development of anxiety and stress in patients. The anxiety scores have remained stable since the mid-1900s, despite improvements in modern dentistry [5]. Our body answers to real or perceived threats, both psychological and physiological. Some of the effects include tachycardia, high blood pressure, hyper-glycemia, mydriasis, hyperthermia, high cholesterol, cortisol secretion. These effects negatively affect important biological mechanisms and is also considered as a risk factor in the development of certain systemic diseases [6, 7,8,9, 10]. Dental anxiety is often associated with postponement or avoidance of dental treatment and, hence, poorer oral health and oral health-related quality of life. Treating such anxious patients requires more treatment time and resources. In addition reduced cooperation ultimately results in an unpleasant experience for both the patient and the dentist [11]. A study by Eli has shown that a strained patient-dentist relationship can lead to misdiagnosis of vitality of pulp [12]. Though there are many ways to identify dental anxiety, in this study it is measured using Modified Dental Anxiety Scale [MDAS]. The questionnaire was asked to be filled in during consultation time. The questionnaire consists of 5 questions grading from 1 to 5. Each question has five different options ranging from a score of 1 [not anxious] to 5 [extremely anxious]. The sum score has a minimum of 5 and a maximum of 25 [13]. Scores 5-9, represents patients who are classified as not dentally anxious or slightly anxious, and thus, not needing any intervention. Scores of patients with higher than 19 are deemed highly anxious, and they might be treated with more complex interventions [14,15]. Patients with scores between 10 and 18 are defined as having moderate dental anxiety. It is found that that individuals with moderate dental anxiety might benefit principally from noninvasive methods, such as listening to music. So patient with moderate dental anxiety score were included in this study. Though there are many strategies followed for coping with stress and emotions such as relaxation, hypnosis and distraction. In recent time music is used to distract the patient which in turn helps to reduce dental anxiety [16]. It has gained popularity due to its non-invasive method. Study by Eleni found that music therapy helps in decreasing the medication [17]. Thus, there is evidence showing that music reduces the negative impact of stress. Music is useful in all age groups to reduce dental anxiety. There are very few literatures on the evaluation of effectiveness of music therapy during dental procedures. A few studies reveal that patients stress level reduced while listening to tamil folk which comes under the genre of popular as people can better understand [17,18]. Extraction has a strong correlation with both pre-treatment dental anxiety and fear of pain during treatment. The subjective reactions by patients to extraction procedures such as local anesthesia, the pressure applied to the tooth during extraction, the tractions and noise recorded, and the time taken to cause anxiety is of interest. Although studies have examined the effects of music therapy in various fields of dentistry, the possibility of dental extraction as a risk factor for altered blood pressure, SpO2 and pulse rate remains unknown. It is a question whether dental extraction could cause a significant change in vital signs which in combination with psychologic and physical stress, painful stimuli that could cause harm or even death to the patient if not managed properly [19, 20] Therefore, it is of interest to report the effect of music on changes in the vital signs and anxiety reactions during dental procedures.

## Materials and Methods:

**Figure 1:**
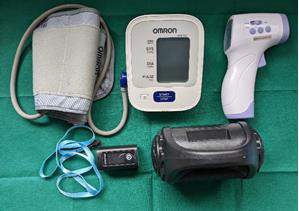
Apparatus used for the study

This study was done in the Department of Dental Surgery, Chengalpattu Government Medical College & Hospital. This study was approved by the Institutional Research Committee and the Institutional Ethical Review Board. Materials used were Omron automatic blood pressure monitor, non-contact infrared thermometer, fingertip pulse oximeter and Zebronics portable speaker as shown in Figure 1. Patients who of 18-60 years, medically fit and indicated for intra-alveolar extraction and exhibiting anxiety based on Modified Dental Anxiety Scale (MDAS) were included in the study. Patients with systemic disorders, mental and physical disability, hearing impairment were excluded. This Randomized control study included 200 patients who were divided into two groups randomly. Music was not played in the control group (n=100) whereas in the experimental group (n=100), music was played according to patients' preference. Blood pressure, Pulse rate, Respiratory rate, Oxygen saturation and Body temperature were recorded using the materials mentioned above a) While waiting in the waiting room b) During the dental treatment c) Post treatment procedure. The values obtained were tabulated on an excel sheet. The respiratory rate was measured manually by observing patient's chest movement.

## Statistical analysis

The values obtained were subjected to statistical analysis. Chi Square test was done to compare Blood pressure. Independent student t test was done to compare other variables. One-way anova was done for within group comparison for all parameters except blood pressure.

## Results:

Statistical difference was found only in respiratory rate which increased during the procedure in both control and experimental groups. There was no significant difference in intra group comparison. However, other vital parameters showed no statistical difference.

## Discussion:

This study was done to see if there is any effect of music over dental anxiety in patients attending a government hospital for tooth extraction towards high quality patient care. Many music theorists such as Bonny [21] and Gfelle [22] have stated that music has the ability to divert the attention from stressful stimuli and to refocus on pleasurable states. Therefore, the current study was carried out in 200 out-patients who suffer from dental anxiety. To begin with, in this study, music chosen by patients was played over the headphones as Spintge suggests self-chosen music is the most effective way to reduce stress. Although using of headphones reduces the impact of the music on staff [23], it does make staff-patient communication more difficult. Whereas, music played over loudspeakers has both positive and negative effects on operating room and over the staff: it can disrupt effective communication, but can also produce a calm working environment [24]. Table 1 shows that there is a significant difference in the blood pressure measured before the procedure between the control and experimental group. The blood pressure in the control group was lesser than that of experimental group. A possible reason could be the confounding factors. As the number of patients with stage II hypertension in the control group was lesser than that of the experimental group. On the other hand, there are no significant changes during and after the procedure. Studies examining the effects of music have had conflicting results. Literature on the effect of music while going through a painful procedure is still inconsistent. De Ramón LA *et al*. [25] stated that music therapy decreased systolic blood pressure, diastolic blood pressure, and heart rate during extraction of impacted third molars. In contrast to that study, Kupeli and Gulnahar [26] found no significant difference between the groups regarding heart rate and mean arterial pressure. They concluded that this could be because of the individual differences in the response of the parasympathetic nervous system during the stimulation of the sympathetic nervous system.

Table 2 reveals that there is no significant difference in the temperature of the patient in all three situations. Similarly, there were no significant changes in oxygen saturation. Though, studies that measure the S_p_O_2_ and pulse rate during routine dental treatment are not common [27,28]. There are studies that have showed the oxygen saturation level remaining stable throughout the dental procedure. This could be due to the duration of the procedure. The average time taken to complete the full procedure was 15 minutes. Furthermore, pulse rate increased during the procedure in both the groups and subsequently decreased after the procedure. The sympathetic nervous system activity tends to increase during dental surgery, because of painful stimuli and psychological stress. This increased sympathetic activity would therefore increase the blood pressure and pulse rate [29]. Although there was a slight rise of pulse rate during the procedure, it was not significant statistically. In addition, it was also evident that respiratory rate was significantly high during the procedure in the intragroup comparison (Table 3) while there was no difference between the control and experimental group. This increase in respiratory rate could be attributed to needle phobia in adult population [30]. Furthermore, they said that clinician's response is not helpful thus concluding that a device needs to be implemented to improve patient's experience.

## Conclusion:

Thus, the current study reveals that the effect of music over a procedure like extraction that has been perceived as painful treatment for a long time in Chengalpattu has little effect on adult patients.

## Clinical Significance:

To reduce the stress factor, though music can be used as one of the aids during extraction procedure at Chengalpattu Hospital, it requires additional aids.

## Figures and Tables

**Table 1:** Comparison of blood pressure before during and after the procedure

**Variables**	**Control Group**	**Experimental group**	**P**
BP - Before			
Low	20	23	
Normal	16	10	
Prehypertension	20	19	0.047*
Stage 1 Hypertension	38	29	
Stage 2 Hypertension	6	19	
BP - During			
Low	15	13	
Normal	24	30	0.118
Prehypertension	40	32	
Stage 1 Hypertension	15	24	
Stage 2 Hypertension	6	1	
BP - After			
Low	9	11	0.314
Normal	27	15	
Prehypertension	33	42	
Stage 1 Hypertension	17	17	
Stage 2 Hypertension	14	15	

**Table 2:** Comparison of temperature in Fahrenheit, pulse rate, SPO2 and respiratory rate before during and after the procedure

**Variables**	**Control Group**	**Experimental group**	**P**
**Temperature**			
Before	97.27±5.82	97.80±0.428	0.3655
During	97.785±1.560	97.32±5.932	0.4641
After	97.272±5.675	97.921±0.424	0.2649
**Pulse rate**			
Before	85.54±11.63	83.87±11.97	0.3045
During	86.20±13.43	84.33±13.19	0.3136
After	83.03±12.89	81.50±13.01	0.3882
**SPO2**			
Before	98.17±0.94	98.32±1.01	0.274
During	98.34±1.07	98.19±1.00	0.2967
After	98.19±1.03	98.04±1.31	0.3547
**Respiratory Rate**			
Before	19.06±3.49	19.14±3.56	0.8723
During	20.81±4.13	20.49±4.08	0.5773
After	19.47±3.75	19.69±3.96	0.6719

**Table 3:** Within group comparison

**Variables**	**Control Group**	**F value**	**P**	**Experimental group**	**F value**	**P**
**Temperature**						
Before	97.27±5.82			97.80±0.43	0.853	0.427
During	97.78±1.56	0.386	0.68	97.32±5.93		
After	97.27±5.67			97.92±0.42		
**Pulse rate**						
Before	85.54±11.63			83.87±11.97		
During	86.20±13.43	1.742	0.177	84.33±13.19	1.422	0.243
After	83.03±12.89			81.50±13.01		
**SPO2**						
Before	98.17±0.94			98.32±1.01		
During	98.34±1.07	0.838	0.433	98.19±1.00	1.576	0.208
After	98.19±1.03			98.04±1.31		
**RR**						
Before	19.06±3.49			19.14±3.56		
During	20.81±4.13	5.804	0.003*	20.49±4.08	3.072	0.048*
After	19.47±3.75			19.69±3.96		
*RR- Respiratory ratev
